# Evaluating and Optimizing Just-in-Time Adaptive Interventions in a Digital Mental Health Intervention (Wysa for Chronic Pain) for Middle-Aged and Older Adults With Chronic Pain: Protocol for a Series of Randomized Trials

**DOI:** 10.2196/77532

**Published:** 2025-09-17

**Authors:** Abby L Cheng, Joanna Abraham, Sarah M Hartz, Eric B Laber, J Philip Miller

**Affiliations:** 1 Division of Musculoskeletal Physical Medicine and Rehabilitation Department of Orthopaedic Surgery Washington University in St. Louis School of Medicine St. Louis, MO United States; 2 Department of Anesthesiology & Institute for Informatics Washington University in St. Louis School of Medicine St. Louis, MO United States; 3 Department of Psychiatry Washington University in St. Louis School of Medicine St. Louis, MO United States; 4 Department of Biostatistics and Bioinformatics Duke University Durham United States; 5 Institute for Informatics, Data Science, and Biostatistics (I2DB) Washington University in St. Louis School of Medicine St. Louis, MO United States

**Keywords:** anxiety, chronic pain, depression, digital mental health intervention, engagement, human-centered design, just-in-time adaptive intervention, microrandomized trial, middle-aged adults, older adults

## Abstract

**Background:**

On a population level, digital mental health interventions effectively reduce depression and anxiety symptoms. However, middle-aged and older adults with chronic pain and coexisting depression or anxiety have not been adequately represented in digital mental health studies.

**Objective:**

The goal of this study is to refine an existing mobile, digital mental health intervention (Wysa for Chronic Pain) that addresses symptoms of depression, anxiety, and coexisting chronic pain for the unique challenges and technology use patterns of middle-aged and older adults.

**Methods:**

Using a mixed methods, human-centered design approach and a series of randomized trials, we will test and iteratively refine just-in-time adaptive interventions (JITAIs) that are designed to increase engagement with a digital mental health intervention. Participants will be aged 45 years or older, endorse at least moderately severe depression or anxiety symptoms (Patient Health Questionnaire-9 or Generalized Anxiety Disorder-7 score ≥10), and have coexisting chronic pain (ie, pain on most days or every day in the past 3 months), and live in the United States. In this open, web-based trial, participants will all receive Wysa for Chronic Pain (by Wysa), which uses a behavioral activation framework and encourages users to work toward pain acceptance. The fully automated intervention also includes cognitive behavioral therapy, mindfulness, and sleep tools, among others. In each trial, participants will be randomized during a maximum 12-week study period to receive versus not receive novel JITAIs that are intended to reduce navigation burden and improve usability (and subsequent engagement and clinical effectiveness). The JITAIs are being designed with iterative user feedback, guided by the Discover, Design/Build, and Test framework and the Behavioral Intervention Technology model. The proximal outcome for each JITAI is related to engagement with Wysa for Chronic Pain after JITAI delivery (compared to when no JITAI is delivered). The primary distal clinical outcome is the Patient Health Questionnaire Anxiety and Depression Scale. Based on statistical analysis that is triangulated with qualitative feedback from a subsample of trial participants, the JITAIs will be iteratively refined and retested in subsequent microrandomized trials until retesting of refined adaptations no longer yields meaningful improvement in immediate engagement or a maximum of 5 total trials have been completed.

**Results:**

Institutional review board approval was obtained on April 11, 2025. The first participant was enrolled on June 2, 2025, and recruitment is expected to conclude in 2026.

**Conclusions:**

Completion of this project will result in iteratively refined JITAIs that are designed to improve usability and engagement with a digital mental health intervention by middle-aged and older adults with depression or anxiety and coexisting chronic pain.

**Trial Registration:**

ClinicalTrials.gov NCT06978166; https://clinicaltrials.gov/study/NCT06978166

**International Registered Report Identifier (IRRID):**

PRR1-10.2196/77532

## Introduction

### Background

The prevalence of chronic pain increases with age, and among the 26% of middle-aged and 31% of older adult Americans with chronic pain, up to half experience depression or anxiety [1-4]. At a population level, digital mental health interventions effectively address many access barriers to mental health care and reduce depression and anxiety symptoms [5-10]. However, middle-aged and older adults with coexisting chronic pain have not been adequately represented in digital mental health intervention studies. This gap is important to address for these populations for multiple reasons: (1) chronic pain reduces the effectiveness of stand-alone mental health treatment unless a person’s pain is simultaneously addressed [11,12]; (2) physical and mental health–related disability in these older age groups has unique downstream effects, such as reductions in the workforce, impact on dependent children, and strain on caregiver availability [13-17]; and (3) use of technology in these age groups is widespread and growing, but these users have unique digital health needs and preferences [5-7,18-21]. Compared to younger users, middle-aged and older adults have unique needs for mental health interventions. For example, they prefer more access to real-time support that supplements automated instructions; they have a greater interest in interventions that address multimorbidity; they prefer different screen layouts due to age-related changes in visual acuity, eye tracking patterns, and dexterity; and they may benefit from more “push” notifications because they check their phones less frequently, and some have age-related cognitive deficits that reduce their ability to independently navigate through apps [18,22-26].

### Wysa for Chronic Pain

Wysa is an established, evidence-based mobile app with over 6.5 million users, and it has the potential to meet the digital mental health care needs of middle-aged and older adults [27,28]. It is available directly to consumers through free and paid subscriptions, and some versions of the platform are covered by select employers and insurers. It delivers evidence-based mental health therapeutic techniques like cognitive behavioral therapy through an artificial intelligence–based chatbot [29,30]. Wysa for Chronic Pain is a specific version of the platform for people with mental health symptoms and coexisting chronic pain [31]. Preliminary studies have demonstrated encouraging signals of effectiveness for Wysa for Chronic Pain among adults across the age spectrum [32-34]. However, middle-aged and older adults specifically identified barriers to engagement related to navigation difficulty within Wysa for Chronic Pain [34].

### Just-in-Time Adaptive Interventions

Just-in-time adaptive interventions (JITAIs) are a unique approach that uses prespecified triggers and leverages user data to deliver a digital intervention, specifically when a person is most in need and receptive to the intervention [35-37]. For instance, a user who is trying to increase physical activity may receive a daily smartphone prompt to encourage physical activity, but only if passive sensors from the smartphone detect that the user is not already active, is not driving or riding in a vehicle, and has not yet met their step goal for the day [38]. Because JITAIs can be pushed to a user when they are most relevant (rather than requiring user-led navigation), JITAIs are particularly appropriate for people with difficulty navigating digital tools. In academic studies, JITAIs have shown promise in driving engagement and improving effectiveness to increase emotional regulation, exercise, and other healthy behaviors; however, the use of JITAIs in commercial digital mental health interventions is relatively novel [39-42].

### Goal of This Study

We are conducting a large-scale, 3-phase project using human-centered design and the Discover, Design/Build, and Test (DDBT) framework to optimize an existing digital mental health intervention (Wysa for Chronic Pain) for middle-aged and older adults with depression or anxiety and coexisting chronic pain [43]. In the first project phase, we conducted semistructured interviews and usability testing among this target user population. During that process, novel JITAIs for Wysa for Chronic Pain were developed using the Behavioral Intervention Technology (BIT) model [36,44], which links established behavior change strategies with technology-related design and implementation factors—for example, timing, medium (text, audio, or video), and complexity of intervention delivery—that affect engagement and subsequent effectiveness of a digital health intervention [44,45].

This study protocol outlines the second project phase. In a series of traditional and microrandomized trials (MRTs) that are interspersed with qualitative feedback from a subgroup of trial participants, the goal of this study is to test and optimize the effectiveness of the novel JITAIs to increase engagement among middle-aged and older adults with Wysa for Chronic Pain. The central hypothesis is that incorporating these JITAIs will increase engagement with Wysa for Chronic Pain by improving usability and, subsequently, will improve depression and anxiety symptoms in this population. After completion of this phase, the third project phase will be a pragmatic, hybrid type 1 effectiveness-implementation trial during which the final JITAIs will be fully integrated into the Wysa for Chronic Pain user experience.

## Methods

### Study Design and Setting

**Figure 1:**
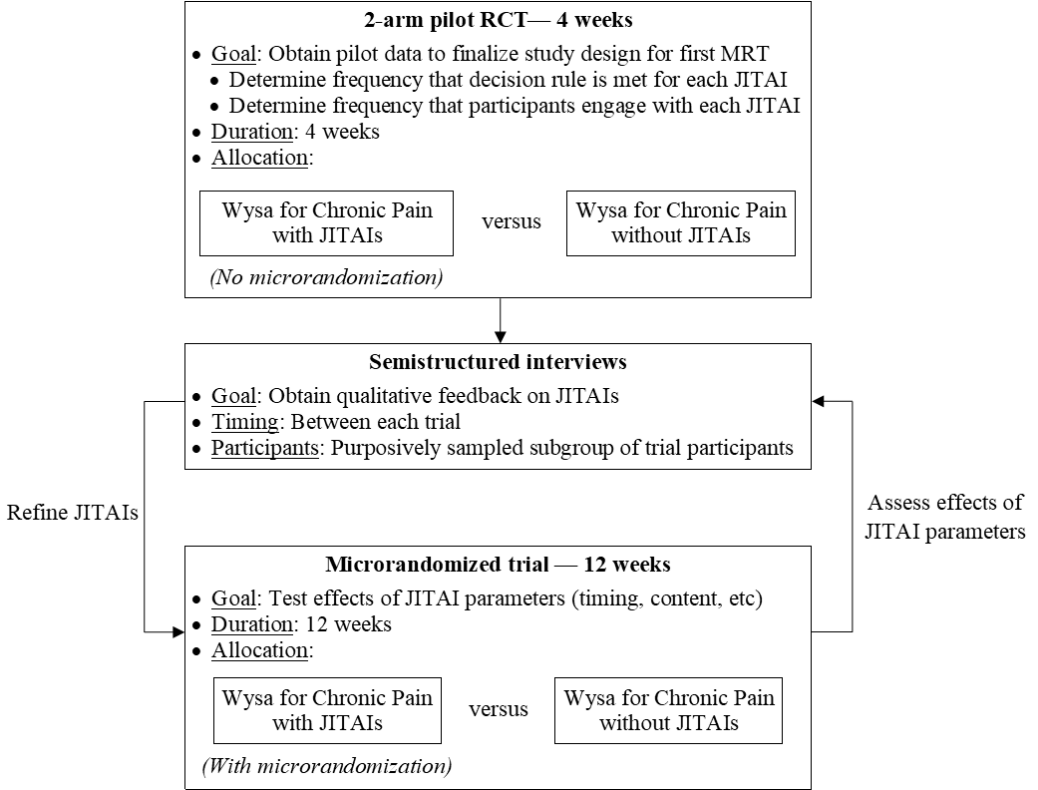
Study design. JITAI: just-in-time adaptive intervention; MRT: microrandomized trial; RCT: randomized controlled trial.

In this remote, single-site, mixed methods study, we will conduct a series of randomized trials interspersed with semistructured interviews (Figure 1). The first trial will be a traditional 4-week, 2-arm randomized controlled trial in which participants receive “Wysa for Chronic Pain with JITAIs” or “Wysa for Chronic Pain without JITAIs.” The primary purpose of this first trial is to obtain preliminary information regarding the frequency that (1) the prespecified criteria (ie, decision rules) are met for each JITAI and (2) participants engage with each JITAI. These quantitative data, coupled with qualitative data from semistructured interviews with a subgroup of trial participants, will guide the final design (and particularly the initial randomization probabilities) of a subsequent 12-week MRT. MRTs were developed as a novel study design by Susan Murphy and colleagues [46] to rigorously study JITAIs. In an MRT, each participant is repeatedly randomized multiple times during the course of the trial. For example, randomization can be related to (1) receiving one versus another JITAI (or none at all) when fixed prespecified criteria are met or (2) variations in the prespecified criteria or delivery of a JITAI (eg, timing for the decision rule, timing of JITAI delivery, JITAI language, etc). In this way, both between-participant and within-participant comparisons can be made, which increases the efficiency of the trial. Furthermore, time-varying effects of a JITAI can be studied with an MRT, for instance, to evaluate a JITAI’s impact on engagement during the first day of app use (when engagement is typically high) compared to the tenth week of app use (when engagement is typically lower) [46].

**Figure 2:**
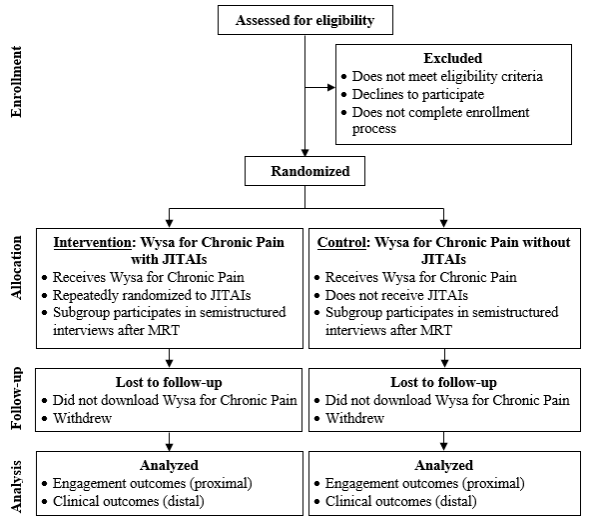
Participant flowchart for each microrandomized trial (MRT). JITAI: just-in-time adaptive intervention.

We anticipate performing 2-4 MRTs as part of this study, depending on opportunities that we identify for continued JITAI refinement based on our quantitative and qualitative findings after each trial, within the limitations of time and budget constraints. Each MRT will include 2 arms (Figure 2). Participants in the “Wysa for Chronic Pain with JITAIs” intervention arm will be randomized every time they meet prespecified criteria (ie, decision rules) for the JITAIs under investigation, whereas participants in the “Wysa for Chronic Pain without JITAIs” control arm will never receive JITAIs, regardless of whether they meet the prespecified criteria. Inclusion of a control arm in each trial will facilitate accounting for time-varying factors (eg, refinements to the Wysa for Chronic Pain user experience that are unrelated to the JITAIs) and enable additional secondary analyses.

### Participants

#### Eligibility Criteria

Eligibility criteria were selected to be pragmatic and reflect the broad target population for Wysa for Chronic Pain while also being mindful of which variables can reliably be collected in an entirely remote study (Textbox 1).

**Eligibility criteria.**

**Inclusion criteria**

- Aged 45 years or older
- At least moderately severe depression or anxiety symptoms (Patient Health Questionnaire-9 or Generalized Anxiety Disorder-7 score ≥10) [47,48]
- Chronic pain (ie, pain on most days or every day in the past 3 months) [2]

**Exclusion criteria**

- Frequent active suicidal ideation [49]
- No access to a mobile device
- Not living in the United States

#### Sample Size

Sample size calculations were performed at a 5% significance level to detect an effect in the primary study outcome between the 2 study arms. Because the primary outcome for each JITAI is related to engagement (which is automatically captured by the Wysa platform and does not require participant report), we assumed a 10% attrition rate (conservatively assessed as occurring at baseline but after downloading Wysa for Chronic Pain). In the initial 4-week pilot trial, we will enroll 29 participants into each of the 2 arms (58 participants total). This will provide 80% power to detect a large effect size (Cohen *d*=0.8).

Sample size determinations for the subsequent 12-week MRTs will be dependent on analysis after each preceding trial, but it is anticipated that control groups for analysis will be pooled by including the control groups from previous trials. For the first MRT, we preliminarily anticipate enrolling 75 participants into the “Wysa for Chronic Pain with JITAIs” intervention arm and 46 participants into the “Wysa for Chronic Pain without JITAIs” control arm (121 total participants recruited). In the intervention arm, clipped Thompson sampling will be used to make intervention assignments with the clipping rate set at 10%. This would provide 80% power to detect a moderate to large effect size (Cohen *d*=0.5). For the second MRT, we preliminarily anticipate enrolling 120 participants into the intervention arm and 55 participants into the control arm (175 total participants recruited). Clipped Thompson sampling will again be used in the intervention arm, with a 10% clipping rate. This would provide 80% power to detect a moderate to small effect size (Cohen *d*=0.4). Sample sizes for any additional MRTs will follow a similar calculation process.

### Randomization

#### Allocation to Interventions

For the initial 4-week pilot trial, a computer-generated random sequence will be used to randomize participants to 1 of the 2 study arms. Stratification (eg, by age, sex, or symptom severity) or block randomization may be considered for the subsequent MRTs based on analysis from the preceding trials. A study statistician will generate the allocation sequences, and participants will be assigned to study arms automatically after they pass the web-based eligibility screen, review the study procedures, and complete the baseline study questionnaires; no direct involvement by study team members is required for study arm assignment. Criteria for discontinuing or modifying allocated interventions are described in Multimedia Appendix 1.

#### Blinding

Study team members involved in outcome assessment and data analysis will not be blinded to participants’ intervention assignment because (1) the primary proximal outcomes related to immediate app engagement will be collected automatically and in real time throughout each trial and (2) the study outcomes will not be analyzed until a given trial is complete. Trial participants cannot definitively be blinded to their intervention assignment because the notification experience within Wysa for Chronic Pain will differ between the 2 intervention arms. Nevertheless, all participants will receive the same Wysa for Chronic Pain therapeutic content, participants will not explicitly be notified which study arm they are assigned to, and participants in the intervention arm with JITAIs will not necessarily be aware when a notification (or lack thereof) from Wysa for Chronic Pain is related to a JITAI under investigation.

### Interventions

#### Wysa for Chronic Pain

**Figure 3:**
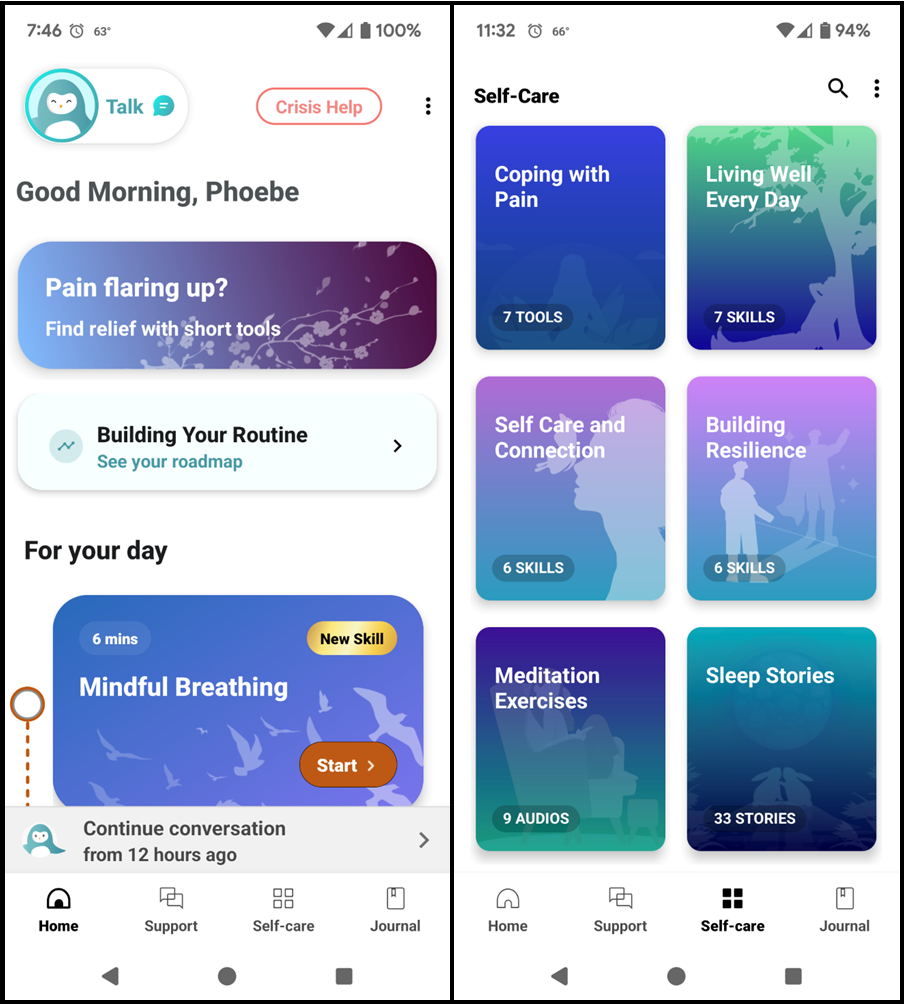
Screenshots of Wysa for Chronic Pain.

The overarching digital intervention is Wysa for Chronic Pain, developed by Wysa for mobile devices (Multimedia Appendix 1). The general Wysa platform delivers cognitive behavioral therapy, dialectical behavioral therapy, motivational interviewing, mindfulness training, deep breathing, and sleep tools (eg, meditations, sleep hygiene education [29,30]) through an artificial intelligence–based chatbot that uses a combination of rule-based decision-making, natural language understanding, and large language models to facilitate maximally nuanced, personalized conversations. Wysa for Chronic Pain also specifically delivers therapeutic content related to behavioral activation and pain acceptance (ie, “acknowledging that one has pain, giving up unproductive attempts to control pain, acting as if pain does not imply disability, and committing one’s efforts toward living a satisfying life despite pain”; Figure 3) [50-53]. It uses push notifications to encourage user engagement and also allows for independent exploration of self-care tools by users. The version of Wysa for Chronic Pain that will be tested in these trials is fully automated.

#### JITAIs Descriptions

Multiple JITAIs will be tested in the trials. Table 1 describes the JITAIs that will be tested in the first trial, but the final plan for each subsequent trial will be refined based on findings from the preceding trials and semistructured interviews. Each proposed JITAI in Table 1 involves the delivery of one or more push notifications to the participant. The notifications are intended to improve engagement in numerous ways, such as directing a user to a relevant therapeutic tool, adjusting the Wysa for Chronic Pain experience and frequency of scheduled notifications in order to meet the user’s demonstrated preferences and to reduce notification fatigue, and increasing the frequency of scheduled notifications when mental health symptoms have recently worsened.

**Table 1:** Description of initial just-in-time adaptive interventions (JITAIs) to be tested in the microrandomized trials.

JITAI name	Purpose	Decision rule: tailoring variables and cut points	Decision rule: intervention^a^
Onboarding	Increase onboarding completion rates	If the user downloaded the app but did not complete onboarding within 24 hours of download	Then, send a notification that encourages the user to finish onboarding. The notification directs the user back to the onboarding screen. When possible, the notification is personalized based on information the user shared thus far during onboarding.
Short disengaged	Motivate a user to engage using messages or content that resonates with the user	If no app engagement for 24 hours	Then, send a personalized notification regarding a challenge the user endorsed at onboarding or during a previous conversation. The notification directly opens to a relevant tool.
Long disengaged	Respect that users’ preferred or needed frequency of engagement may fluctuate based on symptom severity and life events	If no app engagement for 7 days	Then, send an “FYI^b^ or opt back in”^c^ notification, and stop all notifications for 3 days. After 3 days, send a notification with a tool that was previously helpful or that has not been used yet but should be relevant based on challenges reported at onboarding. Then resume the previous notification schedule.
AM check-ins only	Personalize push timing to users’ demonstrated preferences	If no engagement with PM check-ins but ≥1 engagement with AM check-ins within the last 7 days	Then, send an “FYI or opt back in” notification, stop daily “PM check-in” notifications, and move the “activity tracking and mood reflection” task to the AM check-ins.
PM check-ins only	Personalize push timing to users’ demonstrated preferences	If no engagement with AM check-ins but ≥1 engagement with PM check-ins within the last 7 days	Then, send an “FYI or opt back in” notification, stop daily “AM check-in” notifications, and move the “activity scheduling and planning” task to the PM check-ins.
Sporadic engagement only	Personalize push timing and content to users’ demonstrated preferences (eg, engagement only during pain or mood symptom flares)	If no engagement with AM or PM check-ins, but ≥1 user-led engagement within the last 7 days	Then, send an “FYI or opt back in” notification, and stop daily “AM check-in” and “PM check-in” notifications. Instead, each day, send a single personalized notification that directs the user to a tool that was previously helpful or that has not been used yet but should be relevant based on challenges the user reported at onboarding.
Mental health flare^d^	Offer increased support when most needed	If PHQ-4^e^ score worsens compared to the preceding PHQ-4 score, and the user’s current push notification frequency is less than twice daily	Then, send an “FYI or opt out” notification, and increase notification frequency back to twice daily (ie, AM and PM check-ins).

^a^For the “onboarding” and “mental health flare” JITAIs, the intervention will be delivered immediately after the decision rule cut points are met. For all other JITAIs, the intervention will be sent after a strategic delay. If the user’s previous 3 engagement events with the app are within the same 6-hour interval of the day, the initial notification of the intervention will be sent at the time of day equal to the average of the user’s last 3 engagement events (ie, when the user has historically been more likely to engage). Otherwise, the notification will be sent at the last time of day the user entered the app.

^b^FYI: for your information.

^c^If the “opt back in” or “opt out” option is selected, the rest of the intervention is not delivered, the countdown clock for the decision rule resets, and a single personalized notification is sent that directs the user to a tool that was previously helpful or that has not been used yet but should be relevant based on challenges the user reported at onboarding.

^d^The “mental health flare” JITAI overrides any engagement-related JITAIs, which would otherwise reduce the frequency of push notifications to the user.

^e^PHQ-4: Patient Health Questionnaire-4, a 4-item measure to assess depression and anxiety symptom severity [54,55]. Users are prompted to complete the PHQ-4 once weekly as part of a weekly assessment and progress report.

Among participants in the “Wysa for Chronic Pain with JITAIs” intervention arm for each trial, each time they meet prespecified criteria per the JITAIs’ decision rules, they may receive the JITAI. In the initial 4-week pilot trial, they will receive the respective JITAIs described in Table 1 each time they meet the prespecified criteria. In the subsequent MRTs, they will be randomized to receive the respective JITAI, a variation of the JITAI (eg, different timing or content), or no JITAI at all. When the prespecified criteria are not met, participants will not receive a JITAI-related notification or any JITAI-related interventions. Several JITAIs are anticipated to be tested simultaneously in each MRT.

#### Concomitant Care

Participants will not be restricted from engaging in any concomitant care during the trial, including mental health or chronic pain treatments. Participants will be asked to self-report whether (and what types of) medication they take for their mental health or pain at baseline, and they will be asked whether and how their medication regimens have changed at each follow-up time point. They will also be asked to report whether they had any pain procedures or psychiatry, counseling, or physical therapy visits during each follow-up period.

### Outcomes

The purpose of each JITAI is to increase engagement with Wysa for Chronic Pain by improving usability. Therefore, the outcomes of greatest importance in these randomized trials are the proximal outcomes related to engagement (Table 2). All proximal engagement outcomes will be collected automatically for all participants through Wysa platform’s established infrastructure, regardless of whether participants complete follow-up study questionnaires.

**Table 2:** Proximal outcomes for each of the initial just-in-time adaptive interventions (JITAIs) to be tested in the microrandomized trials.

JITAI name			Proximal outcomes		Outcome type		Target engagement time interval	
Onboarding			Completion of onboarding		Binary		24 hours	
**Short disengaged**								24 hours
	Primary outcome	Any engagement^a^		Binary				
	Secondary outcome	Content engagement^b^		Continuous				
**Long disengaged**								4 days
	Primary outcome	Any engagement		Binary				
	Secondary outcome	Content engagement		Continuous				
**AM check-ins only**								7 days
	Primary outcome	Daily engagement^c^ with the “activity tracking and mood reflection” task		Continuous				
	Secondary outcome	Change in content engagement compared to the 7 days preceding		Continuous				
**PM check-ins only**								7 days
	Primary outcome	Daily engagement with the “activity scheduling and planning” task		Continuous				
	Secondary outcome	Change in content engagement compared to the 7 days preceding		Continuous				
Sporadic engagement only			Change in content engagement compared to the 7 days preceding		Continuous		7 days	
Mental health flare			Change in content engagement compared to the 7 days preceding		Continuous		7 days	

^a^“Any engagement” is operationalized as opening the app during the target engagement time interval.

^b^“Content engagement” is operationalized as the total number of tools, conversations, and check-ins the user engaged with during the target engagement time interval.

^c^“Daily engagement” is operationalized as the total number of days the user completed the task of interest during the target engagement time interval.

Distal clinical outcomes will be collected through a self-reported electronic questionnaire. The primary distal outcome will be the Patient Health Questionnaire Anxiety and Depression Scale (PHQ-ADS) [56,57], which is a combination of the Patient Health Questionnaire (PHQ-9) and Generalized Anxiety Disorder-7 (GAD-7) [47,48]. This single measure captures symptoms from both mental health conditions, which Wysa for Chronic Pain is primarily intended to address. Of note, values of these distal clinical outcomes will be the same across all JITAIs tested. In this formative project, analysis of changes in clinical outcomes from baseline to the final follow-up time point will not directly shed insight into the clinical effectiveness of each JITAI but rather will provide insight into the effectiveness of the entire Wysa for Chronic Pain intervention as it is iteratively refined between randomized trials.

In accordance with the National Institute of Mental Health’s (NIMH) experimental therapeutics approach, measures of clinical mechanisms of action will also be collected electronically through self-report (Table 3). We have previously reported signals of mediation by the 3 proposed clinical mediators of behavioral activation, pain acceptance, and sleep quality [53].

**Table 3:** Operationalization of mediators and outcomes.

Construct					Measure
**Clinical mediators**					
	Behavioral activation			BADS-SF^a^ [58]	
	Pain acceptance			CPAQ-8^b^ [59,60]	
	Sleep difficulty			AIS^c^ [61]	
**Outcomes**					
	**Engagement (proximal)**				
		Immediate engagement^d^ (primary outcome)		See Table 2 for JITAI^e^-specific definitions	
		Engagement need^d^		Number of times that the decision rule criteria were met to trigger each JITAI	
		**Engagement dose**			
			Touches	Number of times a user interacted with Wysa for Chronic Pain (eg, opened a notification, navigated through the app, sent a message to the chatbot, etc)	
			Sessions started	Number of times a digital tool session was opened (even if not completed)	
			Sessions completed	Number of times a digital tool session was completed	
		Retention		Number of days from onboarding until the last day of engagement with Wysa for Chronic Pain	
		Engagement content		Number of interactions in which the participant engaged with each of the following tool types: digital cognitive behavioral therapy tools, digital mindfulness and meditation tools, and digital sleep tools	
	**Clinical (distal)**				
		Depressive and anxiety symptoms^f^ (primary outcome)		PHQ-ADS^g^ [56,57] (Combination of PHQ-9^h^ and GAD-7^i^)	
		Disability		WHODAS 2.0^j^	
		Quality of life		Global QOL^k^ [62]	
		Pain interference		BPI^l^, Pain Interference domain [63]	

^a^BADS-SF: Behavioral Activation for Depression Scale-Short Form.

^b^CPAQ-8: Chronic Pain Acceptance Questionnaire-8.

^c^AIS: Athens Insomnia Scale.

^d^These measures will have unique values for each just-in-time adaptive intervention tested. Values for all other measures will be the same across all JITAIs tested.

^e^JITAI: just-in-time adaptive intervention.

^f^PHQ-9 and GAD-7 scores will also be reported separately [47,48].

^g^PHQ-ADS: Patient Health Questionnaire Anxiety and Depression Scale.

^h^PHQ-9: Patient Health Questionnaire-9.

^i^GAD-7: Generalized Anxiety Disorder-7.

^j^WHODAS 2.0: World Health Organization Disability Assessment Schedule 2.0.

^k^Global QOL: Global Quality of Life Scale.

^l^BPI: Brief Pain Inventory.

The primary end point for each MRT will be 12 weeks, which is a common end point for digital mental health trials to assess durability (Table 4) [6]. Because engagement is known to rapidly wane with digital interventions (and to facilitate mediation analyses), patient-reported measures will also be assessed at 4 and 8 weeks.

**Table 4:** Timing of data collection for each microrandomized trial.

Measure			Baseline		Week 4		Week 8		Week 12^a^
Participant characteristics			✓						
Clinical mediators			✓		✓		✓		✓
**Outcomes**									
	Engagement (proximal)			✓		✓		✓	
	Clinical (distal)	✓		✓		✓		✓	

^a^Primary end point.

### Data Collection and Management

#### Qualitative Interviews

Qualitative feedback will be obtained from a subgroup of approximately 5-10 trial participants through semistructured interviews after each trial. Participants will be asked to share perceptions of the strengths, limitations, and suggestions for improvement related to the JITAIs. Participants will be purposively sampled to represent a range of sociodemographic characteristics and engagement patterns. The interviews will occur through secure audio and video recording hosted by a HIPAA (Health Insurance Portability and Accountability Act)–compliant Zoom (Zoom Communications, Inc) platform. Recording files will be stored on the study institution’s encrypted cloud server, Box (Box, Inc).

#### Retention Strategies

To improve retention, participants will be asked to complete the baseline study questionnaires immediately after the recruitment and enrollment process [64]. They will receive electronic gift cards for each time point that they complete all study questionnaires, but participants will not be paid for their level of engagement with the intervention since this is an outcome of interest. Rather, engagement will be monitored for all participants, regardless of whether they complete study questionnaires through the final study time point. A study team member will reach out to a trial participant by phone call, SMS text message, or email for reminders as needed if the participant does not complete electronic study questionnaires despite repeated automated prompts.

### Statistical Methods

#### Primary Outcome

The main purpose of the initial 4-week pilot trial is to determine the initial randomization probability for each JITAI in the first MRT for the “Wysa for Chronic Pain with JITAIs” intervention arm (ie, to construct a robust informative prior, also called a warm start, for each JITAI). We will also perform between-group comparisons of the primary proximal outcome for each JITAI between the intervention versus control arm (ie, comparison of the primary engagement outcome during the target engagement time interval between participants in the intervention arm when they met prespecified criteria and received JITAIs vs participants in the control arm when they met the same prespecified criteria but did not receive JITAIs). In the subsequent MRTs, analysis of the primary proximal outcome for each JITAI will account for both between-group and within-participant comparisons because participants in the “Wysa for Chronic Pain with JITAIs” arm may have met the prespecified criteria for any given JITAI multiple times during the study period. A secondary analysis of each MRT will also be performed to again construct a prior (warm start) for the subsequent MRT, for instance, centered at the estimated optimal JITAI strategy.

#### Interim Analyses

Interim analyses will be performed to ensure the specified randomization probabilities are producing a sufficient frequency of JITAI delivery to facilitate analysis of the primary outcomes, and randomization probabilities may be updated as needed throughout the trials. No interim analyses of the study outcomes will be performed. Given the relatively short duration and low risk of each trial, early stopping of a trial will only be considered if the data safety monitoring board (DSMB) determines that the intervention is contributing to one or more serious adverse events (SAEs), and the risks of continuation of the study outweigh the potential benefits to the enrolled participants. This is because the risk of harm from the intervention is low, and all participants will be receiving the same established therapeutic content. Sustained engagement and treatment effects are important secondary analyses, and early cessation of trials would jeopardize these analyses.

#### Additional Analyses

In a secondary analysis, we will evaluate whether the JITAIs’ effects on engagement vary with time based on the week of the study. The distal clinical outcomes in Table 3 at weeks 4, 8, and 12 will be analyzed using mixed-effects models to test for moderating effects of participant characteristics—for example, age group (middle-aged: 45-64 years vs older adult: 65 years or older), sex, race and ethnicity, depression or anxiety severity [6], and chronic pain duration—and engagement with the Wysa app (ie, total dose, duration, and content) by including these as interaction terms.

We will also estimate optimal JITAIs for the distal clinical outcomes using methods from reinforcement learning [65,66], and we will evaluate the appropriateness of engagement as a surrogate for long-term clinical outcomes by comparing the performance of an optimal JITAI that targets engagement with one that is optimized for the distal clinical outcomes of interest.

Other secondary analyses will include development of models that predict the likelihood that a user will disengage with Wysa for Chronic Pain; descriptive analyses of the estimated optimal JITAI (eg, if, when, and for whom a JITAI should be recommended); and testing for heterogeneity across patients (ie, comparing the performance of a JITAI rule developed from data pooled across all participants to the performance of a rule learned from each participant’s individual trajectory of data).

Our experimental therapeutics model will be assessed by evaluating for associations between engagement with tools related to each therapeutic target (ie, behavioral activation, pain acceptance, and sleep quality), patient-reported improvement in the corresponding target (ie, clinical mediators), and subsequent improvement in distal clinical outcomes. We will also investigate whether and how these associations vary across patient characteristics, such as age, depression or anxiety severity, pain interference severity, and self-reported digital savviness.

#### Missing Data

Once randomized participants download the Wysa for Chronic Pain app and initiate onboarding, proximal engagement outcomes will be automatically collected by the Wysa platform, regardless of how much or even whether a participant engages with Wysa for Chronic Pain during the rest of the study period, so these data points are not expected to be missing. For participant-reported questionnaire data, all questions will be designed as “required,” but participants will have the option to select “prefer not to answer” for sensitive descriptive variables. In these situations, “prefer not to answer” will be treated as a distinct covariate category in the analyses (rather than as missing data) since it will not necessarily be selected at random and may represent a distinct subgroup of participants. Multiple imputation for sequential randomized trials will be used to address missing data, for instance, if a participant does not complete questionnaires at a follow-up time point or withdraws from the study [67].

### Qualitative Analysis

Recordings of participants’ interviews will be professionally transcribed. A hybrid inductive-deductive coding approach will be used, with the BIT model as a framework. At least 2 team members will be involved in thematic analysis. The primary goal of the qualitative analysis is to identify opportunities for refinement of the JITAIs (eg, decision rules, intervention options, intervention language, etc) to improve their usability and effectiveness.

### Data Sharing Plans

Deidentified participant-level data will be shared through the NIMH Data Archive. Access to the dataset will be managed by the NIMH Data Archive.

### Oversight and Monitoring

#### Trial Steering Committee

Collectively, the trial steering committee includes expertise in geriatric mental health, chronic pain, qualitative and implementation research, and biostatistics, with particular expertise in JITAIs and MRTs. One or more members of the committee will meet with Wysa’s project leads at least biweekly on average.

#### Data Safety Monitoring Board

The DSMB consists of 3 members, collectively with expertise in mental health, chronic pain, behavioral interventions, biostatistics, and clinical trials. Led by a chairperson, the DSMB’s role is to review safety data and provide recommendations about starting, continuing, modifying, or stopping the study, taking into account the following considerations: participant safety, achievement of recruitment targets, research team adherence to applicable protocol requirements, and the completeness, quality, and analysis of safety assessments. The DSMB members are independent from the study funder, trial steering committee, and research team, and they are free from conflicts of interest related to the study intervention. The DSMB met before enrollment of the first participant in the first trial and will meet before launching each subsequent trial, or more frequently if new information raises questions regarding safety.

#### Adverse Event Reporting

Study participants will be encouraged to report any real or perceived adverse events to the research coordinator through telephone or email. These instructions will be included on the study information sheet, in addition to other electronic study materials provided to participants. The coordinator will record all reported events in an adverse event log (including the participant’s name, date, and event description), which will be hosted in the study’s secure Research Electronic Data Capture (REDCap) database [68,69]. If a participant expresses thoughts of self-harm with a plan, means, and intent to carry out the plan through communication with a study team member, a member of the study team will call the participant within 1 business day with the goal of ensuring the participant has access to the appropriate crisis resources. The study coordinator will also notify one or more of the study investigators within 2 business days if an SAE is reported directly from a study participant or from someone on behalf of a participant. SAEs include death; hospitalization; suicidal or homicidal action or ideation with a plan, means, and intent to carry out the plan; new psychosis; new substance dependence; or permanently disabling event. Examples of non-SAEs include worsening anxiety or depression symptoms, newly recognized need for professional behavioral health care, and difficulty with intervention technology. Within the Wysa for Chronic Pain platform, Wysa will also track (1) crisis events that are instances when the app detects user input that is suggestive of suicidal or self-harm intent and then the user confirms this thinking on follow-up questioning by the app and (2) instances in which a user navigates to a crisis hotline from within the app. In these instances, the Wysa platform’s standard safety protocols will automatically be implemented and will direct users to national crisis hotlines.

Any serious event that is deemed by the study investigators to be attributable or possibly attributable to the study intervention will be reported to the institutional review board (IRB) within 24 business hours and to the DSMB within 5 business days of the knowledge of occurrence. In accordance with NIMH policy, any deaths or SAEs that are deemed related to study participation will also be reported to the NIMH within 5 and 10 business days, respectively. These actions and the dates of implementation will be recorded in the adverse event log. If an SAE that is attributable to the study occurs, immediate changes to the study consent form and protocol will be made if indicated. Otherwise, each quarter, the study investigators will classify any reported events as “serious” or “nonserious” and as “attributable,” “possibly attributable,” or “nonattributable” to the study intervention.

#### Protocol Modifications

Any proposed major protocol modifications will be approved by the DSMB and IRB and then communicated to relevant parties through updates to the ClinicalTrials.gov registration or direct communication with participants, as relevant.

### Dissemination Plans

Study results will be submitted for publication in peer-reviewed scientific journals and for presentation at academic conferences, as appropriate. Aggregate study results will also be shared through ClinicalTrials.gov. Furthermore, study results will inform the development of a final refined Wysa for Chronic Pain intervention, which will ultimately be available for widespread public use.

### Ethical Considerations

Participants will be recruited from the general direct-to-consumer Wysa platform, potentially as well as from other sources, such as free and publicly available online support groups and research registries (Multimedia Appendix 1). Given the minimal risk of this study, a waiver of written documentation of informed consent will be used. Instead, potential participants will receive a study information sheet that includes all elements of consent and is presented through the study institution’s secure, HIPAA-compliant, web-based REDCap system [68,69] (Multimedia Appendix 2). IRB approval for the study has been obtained by Advarra, Inc (Pro00085039). Participants will receive a US $25 electronic gift card for each time point that they complete all study questionnaires (eg, baseline, week 4, week 8, and week 12; maximum US $100 for full participation in the MRT). Participants who also complete a semistructured interview will receive an additional US $30 gift card.

## Results

This study was funded on March 4, 2024, by the NIMH as part of grant R01MH131989. IRB approval was obtained on April 11, 2025 (protocol version 1: March 26, 2025). The first participant was enrolled on June 2, 2025, and recruitment is expected to conclude in 2026.

## Discussion

### Significance and Directions for Future Research

This study is an intermediate step in the adaptation and dissemination of a digital mental health intervention that is iteratively co-designed with user input, rigorously tested, and then rapidly made available to the target population. After completion of this study, we will conduct a pragmatic, hybrid type 1 effectiveness-implementation study as the final refined version of Wysa for Chronic Pain is launched for widespread use. This process will facilitate real-world assessment of the effectiveness and reach of the intervention, and it will enable rapid, global availability of the intervention for people in need.

We anticipate that the process and results of this study will also lead to more generalizable insights. For instance, our process can serve as a reproducible, precision medicine approach to tailor digital mental health interventions for people with other medical comorbidities that frequently coexist with mental illness (eg, cancer, diabetes, stroke, heart disease, and lung disease) [70]. Additionally, we hope to identify human-centered design insights that are generalizable to other behavior-based digital health interventions for middle-aged and older adults, such as for symptom tracking, medication adherence, and medical visit reminders [71].

### Limitations

A limitation inherent in most trials involving a digital health intervention is that findings from this study will be most generalizable among people who have a base level of comfort and interest in using technology for health-related purposes. Nevertheless, especially as technology use continues to rapidly increase in this large and growing population [20], the results of this study will still be applicable to a meaningful proportion of middle-aged and older adults. For this pragmatic and somewhat formative study, results will also need to be interpreted in the context that (1) participants are permitted to receive additional treatment beyond what is delivered by the digital intervention and (2) multiple secondary outcomes are being assessed, which increases the likelihood of type 1 errors for these outcomes (but not for the singular primary outcome for each JITAI). Finally, as with most behavioral interventions, participants cannot be blinded.

### Conclusions

Completion of this mixed method series of randomized trials and semistructured interviews will result in iteratively refined JITAIs that are designed to improve usability and engagement with a digital mental health intervention by middle-aged and older adults with depression or anxiety and coexisting chronic pain. The methodological approach and the usability-related insights that are identified from this study can also be used for the development and refinement of other digital behavioral interventions for similar populations.

## Appendix

Multimedia Appendix 1 Additional study details.

Multimedia Appendix 2 Study information sheet.

## Abbreviations

BIT: Behavioral Intervention Technology
DDBT: Discover, Design/Build, and Test
DSMB: data safety monitoring board
GAD-7: Generalized Anxiety Disorder-7
HIPAA: Health Insurance Portability and Accountability Act
IRB: institutional review board
JITAI: just-in-time adaptive intervention
MRT: microrandomized trial
NIMH: National Institute of Mental Health
PHQ-9: Patient Health Questionnaire-9
PHQ-ADS: Patient Health Questionnaire Anxiety and Depression Scale
REDCap: Research Electronic Data Capture
SAE: serious adverse event
