# Supplementary material for: Evaluating and Optimizing Just-in-Time Adaptive Interventions in a Digital Mental Health Intervention (Wysa for Chronic Pain) for Middle-Aged and Older Adults With Chronic Pain: Protocol for a Series of Randomized Trials
Source: JMIR Res Protoc. 2025 Sep 17;14:e77532. doi: 10.2196/77532 (PMC12489412; doi:10.2196/77532)
Supplement: Multimedia Appendix 1 [file resprot_v14i1e77532_app1.docx]

# Evaluating and Optimizing Just-In-Time Adaptive Interventions (JITAIs) in a Digital Mental Health Intervention (Wysa for Chronic Pain) for Middle-Aged and Older Adults with Chronic Pain: Protocol for a Series of Randomized Trials

## Multimedia Appendix 1: Additional study details

***Recruitment and ethical considerations***

Participants will be recruited from a variety of sources, some of which are publicly available. Anticipated recruitment sources include: the general direct-to-consumer Wysa platform (among users who are new to the Wysa platform); targeted advertisement in online community support groups for people with mental health symptoms and/or chronic pain (e.g., The Mighty); the Washington University in St. Louis Volunteer for Health open registry; and the National Institutes of Health (NIH) funded, non-profit program to connect researchers and interested potential research participants (ResearchMatch). Other recruitment methods under consideration include: advertisement through established chronic pain and mental health nonprofit associations; advertisements through regional health systems and organizations for older adults [66]; targeted advertisements in social media platforms (e.g., Facebook); and commercial services that facilitate clinical trial recruitment and have robust systems to guard against malicious actors (e.g., Prolific). Recruitment rates and evidence of possible malicious actors will be monitored throughout the study, and recruitment methods will be refined if needed.

All recruitment sources will direct interested potential participants to a secure Research Electronic Data Capture (REDCap) database for more information about the study and to complete a screening questionnaire to determine eligibility [50, 51]. To reduce the potential for malicious actors, potential participants will not be made aware of the precise eligibility criteria when completing the screening questionnaire. Potential participants who are eligible will be immediately directed to a study information sheet within the REDCap platform. Those who are eligible and agree to participate will be immediately directed to the baseline study questionnaires on the same platform and then will immediately receive a unique referral code to Wysa for Chronic Pain after the baseline questionnaires are complete. For the randomized trials, there will not necessarily be any face-to-face interaction between the participants and study team. However, the potential participants will have the opportunity to read the full study information sheet, download a copy of the form (Multimedia Appendix 2), and optionally call or e-mail a study team member with questions to assist in deciding whether to participate. Furthermore, a study team member may call a potential or enrolled participant (or e-mail, and/or text message an enrolled participant who provided consent for these communication methods) to investigate a possible instance of a malicious actor, to troubleshoot any technical issues raised by the participant, and/or to remind them to complete study questionnaires. For the subgroup of participants who are purposively sampled and agree to also participate in semi-structured interviews, web-based teleconferencing will occur between the study participant and a study team member after they complete the randomized trial.

To reduce the likelihood of fraudulent enrollment attempts and malicious actors (e.g., for secondary gain), the REDCap platform will employ spam-protection strategies. Specifically, reCAPTCHA technology will be used, and the research team will monitor for multiple responses from the same Internet Protocol (IP) address and for responses to a “honeypot question,” which is a screening question that is visible to bots but not to human respondents. Wysa’s platform also employs additional hardening measures, including redemption limits on referral codes used for enrollment into this trial. These strategies minimize the risk of a single malicious actor completing multiple trial enrollments using multiple identities.

The study information sheet clearly explains that the intervention provided in the study is not designed to be used in place of in-person mental health treatment, and potential participants who report frequent active suicidal ideation on the online eligibility screening questionnaire will be automatically and immediately provided with contact information for national crisis help lines. Additionally, all study team members will have completed Collaborative Institutional Training Initiative (CITI) and Good Clinical Practices (GCP) training and will have training in the study’s safety protocols. If a potential or enrolled participant expresses active thoughts of self-harm or other mental health crisis to a study team member, the participant will be directed to a national crisis help line. Furthermore, within Wysa for Chronic Pain, all participants will be provided crisis resources (including the United States 988 national crisis hotline) and the opportunity to create a personalized safety plan with contact information for trusted people and places. Additionally, Wysa has built-in technology and protocols for the chatbot to recognize users in crisis who are at risk of harm to themselves or others. If crisis is suspected, the chatbot automatically refers the user to the crisis resources.

All recruitment, eligibility screening, and consent materials will be written in English and indicate that researchers from the Washington University in St. Louis School of Medicine are conducting the study.

***Wysa for Chronic Pain***

The overarching digital intervention is Wysa for Chronic Pain, developed by Wysa for mobile devices. The original Wysa platform was developed in 2016 and delivers cognitive behavioral therapy, dialectical behavioral therapy, motivational interviewing, mindfulness training, deep breathing, and sleep tools (e.g., meditations, sleep hygiene education [29, 30]) via an artificial intelligence based chatbot. Generative artificial intelligence using external large language models (LLMs) powered by OpenAI is used to optimize the conversation experience by facilitating maximally nuanced, personalized conversations within Wysa. Licensed psychologists and conversational designers were involved in content development.

In 2020, Wysa developed a version of the platform called Wysa for Chronic Pain specifically for people with mental health symptoms and coexisting chronic pain [31]. In addition to offering Wysa’s standard features, Wysa for Chronic Pain delivers therapeutic content related to behavioral activation and pain acceptance (i.e., “acknowledging that one has pain, giving up unproductive attempts to control pain, acting as if pain does not imply disability, and [committing] one’s efforts toward living a satisfying life despite pain”) [52-55]. Wysa for Chronic Pain was further refined using iterative rounds of semi-structured interviews and usability testing by middle-aged and older adults who have chronic pain and coexisting symptoms of depression and/or anxiety. The current version of Wysa for Chronic Pain directs users through a predetermined eight-week curriculum, while also allowing for additional exploration of its tool library at any time. After eight weeks, users choose whether to continue the curriculum-based structure versus transition to fully independent navigation of the tool library. By default, push notifications are delivered to users twice daily, once in the morning to schedule/plan an activity and complete a therapeutic tool, and once in the evening to assess completion of the activity, reflect on the user’s associated mood, and complete a sleep tool. The frequency of additional push notifications will vary based on participants’ use patterns and their randomization status for each JITAI under investigation. Wysa for Chronic Pain provides a weekly report with feedback to users regarding their engagement and self-reported progress in relation to the tools they used. Wysa for Chronic Pain is intended to be able to function as a self-care support tool, although it can also be incorporated as a supplementary component to traditional face-to-face psychotherapy and/or pharmacologic management.

For this study, participants will receive access to all features of Wysa for Chronic Pain at no charge. Aside from prompts delivered as part of the clinical intervention and JITAIs under investigation, no additional prompts to encourage engagement will be delivered purely for the sake of the study. The platform will be delivered in English. During the trials, any critical bug fixes that are essential to successful completion of the trial will be implemented immediately. Non-critical revisions and software updates to Wysa for Chronic Pain are expected to occur in between trials based on quantitative results and semi-structured interviews with a subgroup of trial participants.

***Criteria for discontinuing or modifying allocated interventions***

Modifications to JITAIs and/or creation of novel JITAIs are expected to occur between MRTs in response to results of the preceding MRT and of the qualitative feedback from a subgroup of trial participants. Wysa will build these adaptations into its platform, and the adaptations will be tracked using the Framework for Reporting Adaptations and Modifications-Enhanced (FRAME) [72]. No modifications to JITAIs are anticipated during a randomized trial unless there is a concern for safety. Modifications to the randomization probability for each JITAI (for the “Wysa for Chronic Pain + JITAIs” intervention arm) will occur in a batch learning approach.

***Data management and confidentiality***

All quantitative data will either be collected automatically within Wysa’s secure platform or entered directly by participants into Wysa’s platform or the secure REDCap database. The Wysa platform used for this project is HIPAA- and GDPR-compliant. It uses field-level encryption and complies with industry-standard encryption protocols including TLS.1 and AES-256 for data in motion and at rest. Wysa’s platform is certified for ISO/IEC 27001: 2022 and ISO/IEC 27701: 2019. User conversations within Wysa are anonymized before sharing with external LLMs, are deleted by external LLM servers within 30 days, and are not used by external LLMs for internal or external purposes. Wysa will perform continuous, real-time data monitoring during the trials via automated reports in order to limit threats to data quality due to any technical glitches [66]. Wysa’s databases are deployed across at least three geographically separated data centers and have active-active redundancy with near-instantaneous failover.

The data manager from the study team will receive raw participant data from Wysa and will store it in the study institution’s encrypted cloud server, Box. The data manager will perform range and consistency checks to ensure data quality. The Wysa data files will be linked to individual participants via a randomly generated unique identifier. Only research team members at the study institution will have access to participants’ personally identifiable information which can be linked with their Wysa engagement data.
