# Supplementary material for: Evaluating and Optimizing Just-in-Time Adaptive Interventions in a Digital Mental Health Intervention (Wysa for Chronic Pain) for Middle-Aged and Older Adults With Chronic Pain: Protocol for a Series of Randomized Trials
Source: JMIR Res Protoc. 2025 Sep 17;14:e77532. doi: 10.2196/77532 (PMC12489412; doi:10.2196/77532)
Supplement: Multimedia Appendix 2 [file resprot_v14i1e77532_app2.docx]

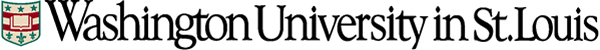


**INFORMED CONSENT FORM
AND**

**AUTHORIZATION TO USE AND DISCLOSE PROTECTED HEALTH INFORMATION**

| **Sponsor / Study Title:** | **Washington University School of Medicine / “Evaluating and Optimizing Just-In-Time Adaptive Interventions (JITAIs) in a Digital Mental Health Intervention (Wysa for Chronic Pain) for Middle-Aged and Older Adults with Chronic Pain”** |
| --- | --- |
| **Protocol Number:**  **HRPO #:** | **WUWYSA02**  **202502047** |
| **Principal Investigator:** | **Abby Cheng, MD, MPHS** |
| **Telephone:** | **314-747-8489 (24 Hours)** |
| **Address:** | **Washington University School of Medicine**  **660 S Euclid**  **Campus Box 8233**  **Saint Louis, MO 63110** |

**Fast Facts**

**Why is this study being done?**

The purpose of this research study is to refine customized in-app notifications in order to optimize users’ experience with a mobile app called Wysa for Chronic Pain. This app is designed to support people who have chronic pain and who also experience symptoms of depression and/or anxiety. It is not currently available to the public. The National Institutes of Health is funding this research study through Washington University.

**What will I do if I participate in the study?**

If you are eligible and choose to participate, you will be asked to download and use the Wysa for Chronic Pain study app for 4 weeks. You should use the study app as you normally would, as if you were not part of a research study – use it as much and as often as is helpful for you and works with your schedule. Note: this study app is not designed to be used in place of in-person mental health treatment. You will also be asked to complete 10-15 minute surveys about your mood, pain, physical function, and sleep at the beginning of study and then again 4 weeks later.

All participation will take place on the study app, and the surveys will be filled out on Washington University's secure study database (called REDCap). If you have not responded to the surveys within two days, the research team will send up to three text reminders to the e-mail address or phone number that you provide and may follow up with reminder phone calls. There are no in-person visits. Approximately 56 people will take part in this study.

Additionally, about 5 of the participants will be asked to share their experience with the study app at the end of the 4 weeks in a casual interview using a secure, Health Insurance Portability and Accountability Act (HIPAA)-compliant Zoom audio/video recorded call. You may decline this interview portion if you are invited to do it.

**Am I eligible for this study?**

To determine if you are eligible for this research study, we need to ask a few questions about your recent mood and pain. After reading the additional study information below, please fill out the survey starting at the bottom of this page.

If you are eligible, you will be directed to the next steps. Even if you are eligible, participation in this research study is voluntary. You may choose to participate or not. If you are not eligible, you will be notified when you finish the eligibility survey.

**Additional Information**

**Risks and Benefits**

If you participate in this study, the main risk to you is that confidential information about you may be accidentally disclosed. We will use our best efforts to keep the information about you secure. There may be risks that are unknown.

If you participate, you may benefit by receiving access to the Wysa for Chronic Pain study app, which is not currently available to the public. We also hope that other people may benefit in the future from what we learn from this study.

**Payment/Compensation for Participants**

You will not have any costs for being in this research study.

You will be paid for being in this research study. A $25 gift card to one of several businesses will be delivered to you upon downloading/activating the study app and completing the research surveys at the beginning of the study and 4 weeks later, for a total of $50 if you complete the surveys at both time points. You can choose for your gift cards to be delivered via e-mail or postal mail.

Additionally, if you are one of the 5 people who share their experiences with the in-study app notifications at the end of the study in a casual interview, you will be paid another $30 gift card for the completion of that audio/video recorded interview. You may decline that portion if you are invited to it.

You will be paid following each completed visit.

Compensation for participation in research may be considered taxable income. Washington University requires tracking for compensation that is paid to you; this may include your name, contact information, and social security number for reporting purposes. It is possible to receive compensation without providing your social security number. You would need to provide your mailing address if a gift card will be mailed to you, or an e-mail address to send the gift card electronically.

**Data Sharing**

The data we are obtaining in this study may be made available for studies going on right now, as well as for studies that are conducted in the future. These studies may be done by researchers at Washington University, other research centers and institutions, or private companies involved in research. We may also share your research data with large data repositories, such as the National Institute of Mental Health Data Archive (NDA). A repository is a database of information for use by others, such as the research community, institutions, private companies, and the public. If your individual research data is placed in one of these repositories, your name and other identifying information will be removed. All reasonable precautions will be taken to protect your privacy and confidentiality. Necessary approvals will be obtained to use the data. Certain summary information may be available to the public. Your data will be stored without your name or any other kind of link that would enable us to identify which data are yours. Therefore, it will be available indefinitely for use in future research studies without your additional consent and cannot be removed.

You may decide that you don’t want your study data to be included in the data repositories. You can still participate in this research study even if you decide that you do not want your data to be added to the data repository.

**Audio Recording/Video Recording**

Some participants will be asked to share their experiences with the in-study app notifications using audio and video recording via secure Zoom-HIPAA technology. The recordings will be used to transcribe the interview conversation to accurately remember what you say during the study. After the interview, the recording will be reviewed by a research team member, and all personal identifiers will be removed prior to being uploaded into a secure server to be transcribed into text for analysis. Audio recording is required for this part of the study so that we can create an accurate transcript of what you say. While all recordings are stored in a confidential manner, please be aware that it may be possible to identify you from your voice recording.

For participants who are asked and choose to take part in the interview portion of the study, video recording may be required, at least for a brief part of the interview. Participation in the video recording part helps us be as certain as possible that we accurately remember what you say and mean during the study. The video recording will include facial features that can be used to identify you. It is possible that your face may be recognizable, but the video recording will not be shared with anyone besides research team members.

**Alternatives to Participation**

This research study is for research purposes only. The only alternative is to not participate in this study.

**Injury Language**

Washington University investigators and staff will try to reduce, control, and treat any complications from this research. If you feel you are injured because of the study, please contact the investigator at the telephone number listed on the first page of this form and/or the Human Research Protection Office at 1-(800)-438-0445.

Decisions about whether payment for medical treatment for injuries relating to your participation in research will be made by Washington University. If you need to seek medical care for a research-related injury, please notify the investigator as soon as possible.

**How Will You Keep My Information Confidential?**

Other people such as those listed below may become aware of your participation in this study and may inspect and copy records pertaining to this research. Some of these records could contain information that personally identifies you.

- Government representatives (including the Office for Human Research Protections) to complete federal or state responsibilities
- National Institutes of Health
- The sponsor (National Institutes of Health) may also inspect any part of your medical record for the purposes of auditing the conduct of the study.
- Hospital or Washington University representatives to complete their responsibilities
- Information about your participation in this study may be documented in your health care records and will be available to anyone with access to your health care record, including your health insurance company. This information may also be released as part of a release of information request.
- Washington University’s Institutional Review board (a committee that oversees the conduct of research involving human participants) and the Human Research Protection Office
- Advarra IRB **(**An Institutional Review Board has reviewed and approved this study).

Any report or article that we write will not include information that can directly identify you. The journals that publish these reports or articles require that we share your information that was collected for this study with others to make sure the results of this study are correct and help develop new ideas for research. Your information will be shared in a way that cannot directly identify you.

Protected Health Information (PHI) is health information that identifies you. PHI is protected by federal law under HIPAA (the Health Insurance Portability and Accountability Act). To take part in this research, you must give the research team permission to use and disclose (share) your PHI for the study as explained in this consent form. The research team will follow state and federal laws and may share your health information with the agencies and people listed above.

Once your health information is shared with someone outside of the research team, it may no longer be protected by HIPAA and may further be shared without your permission.

The research team will only use and share your information as talked about in this form or as permitted or required by law. When possible, the research team will make sure information cannot be linked to you (de-identified). Once information is de-identified, it may be used and shared for other purposes not discussed in this consent form. If you have questions or concerns about your privacy and the use of your PHI, please contact the University’s Privacy Officer at 866-747-4975.

Although you will not be allowed to see the study information, you may be given access to your health care records by contacting your health care provider.

**If you decide not to sign this form, it will not affect**

- your treatment or the care given by your health provider.
- your insurance payment or enrollment in any health plans.
- any benefits to which you are entitled.

However, it will not be possible for you to take part in the study.

**If you sign this form:**

- You authorize the use of your PHI for this research
- This authorization does not expire.
- You may later change your mind and not let the research team use or share your information (you may revoke your authorization).
- To revoke your authorization, complete the withdrawal letter, found in the Participant section of the Human Research Protection Office website at [http://hrpo.wustl.edu/participants//withdrawing-from-a-study/](http://hrpo.wustl.edu/participants/withdrawing-from-a-study/) or you may request that the investigator send you a copy of the letter.
  - **If you revoke your authorization:**
    - The research team may only use and share information already collected for the study.
    - Your information may still be used and shared as necessary to maintain the integrity of the research, for example, to account for a participant’s withdrawal from the research study or for safety reasons.
    - You will not be allowed to continue to participate in the study.

We will keep the information you provide confidential by keeping all electronic data in a password protected database that is only accessible to research team members. However, federal regulatory agencies, Washington University, and the Advarra Institutional Review Board (which is a committee that reviews research studies) may inspect and copy records pertaining to this research. This means that absolute confidentiality cannot be guaranteed. If we write a report about this study, we will do so in such a way that you cannot be identified. The funding source for this research may require that we share the data from this study with others to make sure the results are correct and to use for future research. Your information will be shared in a way that cannot directly identify you.

To further protect your privacy, this research is covered by a Certificate of Confidentiality from the federal government. This means that the researchers can refuse to disclose information that may identify you in any legal or court proceeding or to anyone who is not connected with the research except if:

- There is a law that requires disclosure, such as to report child abuse and neglect, or harm to self or others*;*
- You give permission to disclose your information, including as described in this consent form; or
- It is used for other scientific research allowed by federal law.

This Certificate may not be effective for information held in foreign countries. You have the right to share your information or involvement in this study with anyone at any time. You may also give the research team permission to disclose your information to a third party or any other person not connected with the research.

When possible, the research team will make sure information cannot be linked to you. Once information doesn't identify you, it may be used and shared for other purposes not discussed in this document.

A description of this clinical trial will be available on http://www.ClinicalTrials.gov, as required by U.S. Law. This Web site will not include information that can identify you. At most, the Web site will include a summary of the results. You can search this Web site at any time.

**Voluntary Participation**

Your participation in this study is completely voluntary. You may choose not to take part at all.  If you decide to participate in the study, you may stop participating at any time. Any data that was collected as part of this study will remain as part of the study records and cannot be removed.  If you decide not to take part in the study or if you stop participating at any time, you won't be penalized or lose any benefits for which you otherwise qualify.

Any new important information that is discovered during the study and which may influence your willingness to continue participation in the study will be provided to you.

The Investigator or the sponsor can stop your participation at any time without your consent for the following reasons:

- If it appears to be medically harmful to you;
- If you fail to follow directions for participating in the study;
- If it is discovered that you do not meet the study requirements;
- If the study is canceled; or
- For administrative reasons.

**Whom to Contact About This Study**

During the study, if you experience any medical problems, suffer a research-related injury, or have questions, concerns or complaints about the study such as:

- Whom to contact in the case of a research-related injury or illness;
- Payment or compensation for being in the study, if any;
- Your responsibilities as a research participant;
- Eligibility to participate in the study;
- The Investigator’s or study site’s decision to withdraw you from participation;
- Results of tests and/or procedures;

**Please contact the Investigator at the telephone number listed on the first page of this consent document.**

If you seek emergency care, or hospitalization is required, alert the treating physician that you are participating in this research study.

An institutional review board (IRB) is an independent committee established to help protect the rights of research participants. If you have any questions about your rights as a research participant, contact:

- By **mail**:

Study Subject Adviser

Advarra IRB

6100 Merriweather Dr., Suite 600

Columbia, MD 21044

- or call **toll free**:    877-992-4724
- or by **email**:          [adviser@advarra.com](mailto:adviser@advarra.com)

Please reference the following number when contacting the Study Subject Adviser: Pro00085039.

You may also contact the Human Research Protection Office at 660 South Euclid Avenue, Campus Box 8089, St. Louis, MO 63110, 1-(800)-438-0445, or email hrpo@wusm.wustl.edu. General information about being a research participant can be found on the Human Research Protection Office web site, http://hrpo.wustl.edu. To offer input about your experiences as a research participant or to speak to someone other than the research staff, call the Human Research Protection Office at the number above.

Thank you very much for your consideration of this research study.

*Filling out the form below signifies that you understand the nature of this research study and you wish to see if you are eligible to participate. By filling out this form, you agree to share your answers with the research team. (You will not be paid for completing the eligibility survey.)*

- Do you allow us to send study information (like survey links) to your e-mail address?

(If someone reads the e-mail, they may know that you are participating in this research study.)

__Yes, okay to send me survey links using e-mail __No

- Do you allow us to send study information (like survey links) to you via text message (SMS)?

(If someone reads the text message, they may know that you are participating in this research study.)

__Yes, okay to send me survey links via text message __No

Messages will come from 833-858-5729: consider saving this number in your phone. Standard data rates may apply. Up to 3 text message reminders would be sent for each survey. You may reply STOP to opt out.

**Do you give permission for your data to be used in future research studies and stored in a large data repository after your personal identifying information (such as name, birthdate, etc.) has been removed?**

(You can participate in this research study even if you do not give permission for your data to be used in future research studies.)

__Yes, okay to use my data in other research __No

studies after my personal information has been removed

**To review:**

**If you agree to participate in this study, you will be asked to:**

1. **Download the Wysa for Chronic Pain study app (for free).**
2. **Use the Wysa for Chronic Pain study app for the next 4 weeks, whenever and however it is helpful to you.**
3. **Complete a 10-15 minute survey now, and again in 4 weeks.**

I have read and understand the information in this informed consent document. I have had an opportunity to ask questions and all of my questions have been answered to my satisfaction. I voluntarily agree to participate in this study until I decide otherwise. I do not give up any of my legal rights by signing this consent document. I will receive a copy of this signed consent document.

**By clicking “I want to enroll” and continuing to the next page, you are indicating that this research study has been explained to you, that your questions have been answered, and that you agree to take part in this study.**
